# BioCode gold-nanobeacon for the detection of fusion transcripts causing chronic myeloid leukemia

**DOI:** 10.1186/s12951-016-0192-y

**Published:** 2016-05-17

**Authors:** M. Cordeiro, L. Giestas, J. C. Lima, P. M. V. Baptista

**Affiliations:** LAQV, REQUIMTE, Departamento de Química, Faculdade de Ciências e Tecnologia, Universidade Nova de Lisboa, Campus de Caparica, 2829-516 Caparica, Portugal; CIGMH, UCIBIO, Departamento de Ciências da Vida, Faculdade de Ciências e Tecnologia, Universidade Nova de Lisboa, Campus de Caparica, 2829-516 Caparica, Portugal

**Keywords:** Gold nanoparticles, FRET, Hybridization, Biosensor, BCR-ABL fusion, Leukemia

## Abstract

**Background:**

Gold-nanobeacons (Au-nanobeacons) have proven to be versatile systems for molecular diagnostics and therapeutic actuators. Here, we present the development and characterization of two gold nanobeacons combined with Förster resonance energy transfer (FRET) based spectral codification for dual mode sequence discrimination. This is the combination of two powerful technologies onto a single nanosystem.

**Results:**

We proved this concept to detect the most common fusion sequences associated with the development of chronic myeloid leukemia, e13a2 and e14a2. The detection is based on spectral shift of the donor signal to the acceptor, which allows for corroboration of the hybridization event. The Au-nanobeacon acts as scaffold for detection of the target in a homogenous format whose output capability (i.e. additional layer of information) is potentiated via the spectral codification strategy.

**Conclusions:**

The spectral coded Au-nanobeacons permit the detection of each of the pathogenic fusion sequences, with high specificity towards partial complementary sequences. The proposed BioCode Au-nanobeacon concept provides for a nanoplatform for molecular recognition suitable for cancer diagnostics.

**Electronic supplementary material:**

The online version of this article (doi:10.1186/s12951-016-0192-y) contains supplementary material, which is available to authorized users.

## Background

Gold nanoparticles (AuNPs) have been proposed as effective platforms for the development of bio-sensing systems, due to their ease of synthesis, surface functionalization, high surface-to-volume ratio and size-dependent optical properties [1–3] in particular the ability to modulate the emission of nearby organic fluorophores in a distance dependent manner [4–7]. Au-nanobeacons are AuNPs functionalized with a single strand DNA (ssDNA) with a hairpin structure, harboring a fluorophore in the opposite extremity from the AuNP surface [8–10]. Due to their localized surface plasmon resonance (LSPR), AuNPs exhibit a high extinction coefficient (normally three orders of magnitude higher than conventional fluorophores [11]) and may act as a dark quencher upon which multiple hairpins may be grafted. In absence of a complementary target, the hairpin is in its close conformation, keeping the fluorophores in close vicinity of the AuNP, and quenching of the emission is observed. Presence of a complementary target opens the hairpin structure and the fluorophores part away from the AuNPs’ surface, allowing for partial fluorescence recovery [9]. This Au-nanobeacon concept has been used for specific and selective sequence recognition with a diversity of applications [8, 9, 12–14]. Here we present a hybridization based biosensor concept that combines spectral codification with the Au-nanobeacon technology—BioCode Au-nanobeacon. This conceptual biosensor was developed and optimized for the detection and discrimination of similar sequences, such as fusion genes and/or splice variants. This BioCode Au-nanobeacon is labeled with a fluorophore that ay act as a donor for Forster resonant energy transfer (FRET) to an acceptor fluorophore on a second oligonucleotide sequence that, in its turn, will hybridize in the vicinity of the donor upon target recognition. This event will generate a characteristic FRET signal enabling the evaluation of a sample’s composition in terms of presence/absence of target sequence [15, 16]—spectral codification. The specific spectral signature of donor and acceptor pairs formed provides an additional level of information of the hybridization events occurring in solution [17, 18].

The effective sequence selectivity of the BioCode Au-nanobeacon was demonstrated using the most common BCR-ABL fusion transcripts (e13a2 and e14a2) as a model. The formation of the BCR-ABL fusion sequences is dependent on the reciprocal translocation between chromosome 9 and 22, originating the Philadelphia chromosome (Ph + chromosome), which is the molecular hallmark of chronic myeloid leukemia (CML) [19, 20]. This translocation generates very similar mRNA sequences to those on the normal (non-fused) chromosomes (ABL and BCR genes). Therefore, the resultant transcripts may be used for evaluating the discrimination capability of the proposed biosensor.

Here, we report the conceptual development of the *BioCode Au*-*nanobeacon* strategy and demonstrate its application towards discrimination of two fusion genes isoforms responsible for chronic myeloid leukemia.

## Results and discussion

### Hairpin design and specificity assessment for FRET based Au-nanobeacon

CML is the result of a reciprocal translocation between human chromosome 9 and 22, resulting in a fusion gene BCR-ABL that induces cancer cell progression [21]. For the BioCode Au-nanobeacon assembly, the loop portion of the hairpin structure was designed to specifically detect either the e13a2 or the e14a2 BCR-ABL fusion sequence [20, 21]. The palindrome portion (responsible for the closed structure of the ssDNA—hairpin conformation) was designed to have a lower melting temperature (nearest neighbor estimation, nnTm) [22] than that of the double strand formed between the loop portion and the fusion sequence BCR-ABL (target). Hybridization between the different sequences was predicted using the software package NUPACK [23, 24]—see Additional file 1: Figure S1. The reasoning behind the Au-nanobeacon configuration is that the AuNP acts both as a scaffold (multiple hairpins on a single platform heightening local concentration of recognition probes) and as dark quencher for the donor.

**Scheme 1:**
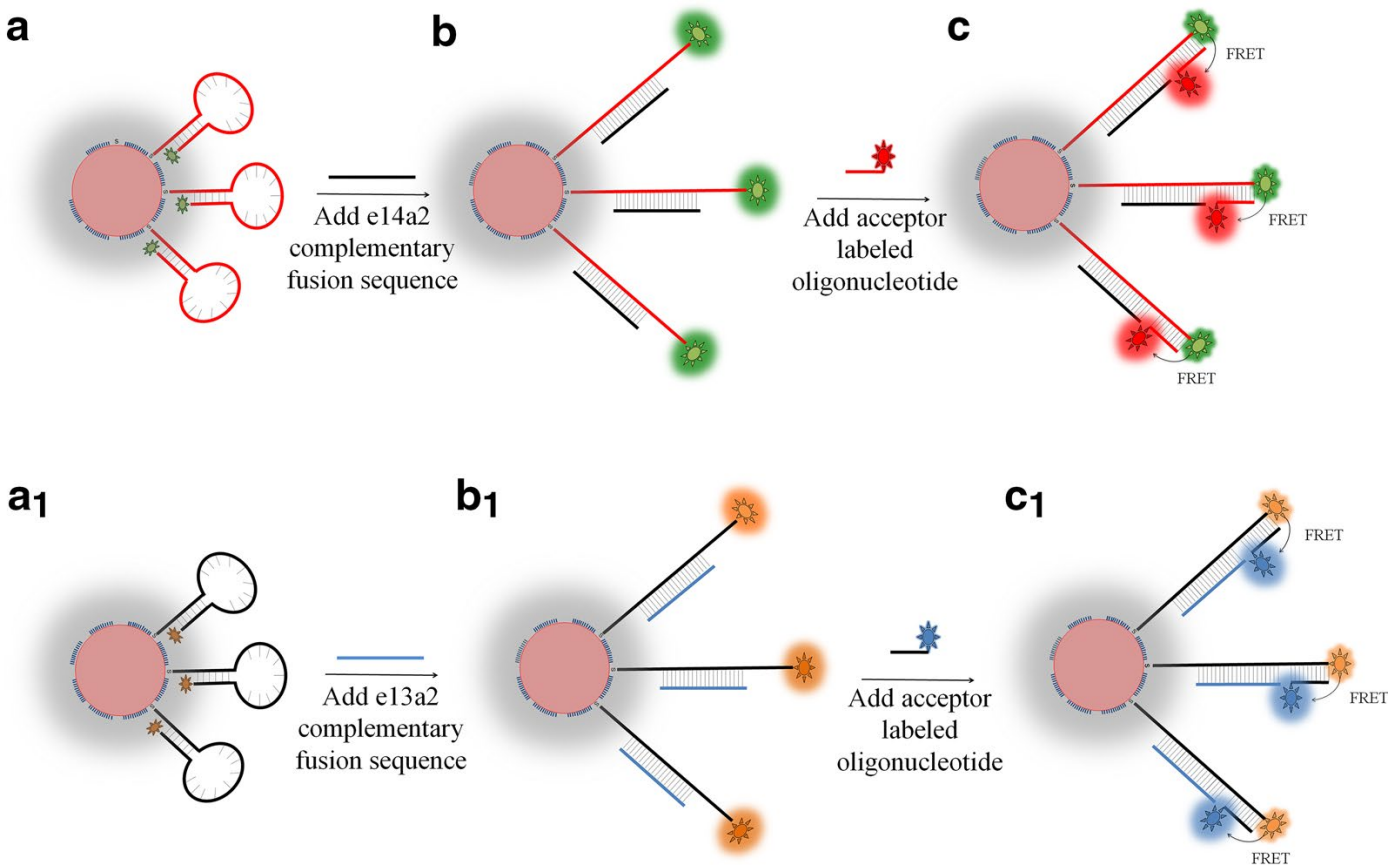
Schematic representation of the BioCode Au-nanobeacon. **a** The hairpin in its closed conformation strongly suppresses fluorescence. **b** A complementary target disrupts the hairpin and the donor breaks away from the AuNP surface leading to partial recovery of the donor’s fluorescence. **c** Hybridization to the target sequence exposes the palindromic sequence, which can then be targeted by the acceptor labeled oligonucleotide triggering the emission of fluorescence signal due to the specific FRET between the fluorophores that codify each possible target sequence (spectral codification)

The BioCode Au-nanobeacon system relies on the FRET mediated talk between fluorophores on the hairpin (surface of AuNPs) and the acceptor labeled oligonucleotides. The working principle of the BioCode Au-nanobeacon is depicted in Scheme 1. In the closed state, the donor is quenched by its proximity to the AuNP surface—donor quenching (Scheme 1a, a_1_); upon hybridization to the target (BCR-ABL fusion product), the hairpin opens, increasing the distance between donor and the AuNP surface causing a partial recovery of fluorescence emission (Scheme 1b, b_1_). Once the palindromic sequence is exposed, the acceptor labeled oligonucleotide hybridizes to it allowing FRET between both fluorophores to occur (Scheme 1c, c_1_), leading to signal output.

Hybridization of the complementary target (either e13a2 OR e14a2 fusion sequences—see Additional file 1: Figure S2) and non-complementary or partial complementary target (ABL and BCR normal expressed genes) was experimentally assessed prior to AuNP functionalization. In these experiments, the expected FRET signal was only generated in the presence of the fully complementary target, demonstrating that lack of full target complementarity does not lead to hairpin opening (see Additional file 1: Figure S3). As such, these hairpin sequences were used to functionalize the AuNPs.

### BioCode Au-nanobeacon synthesis

The synthesized AuNPs presented an average diameter of 14.6 ± 1.7 nm, whose surface was covered at 45 % with thiolated polyethylene glycol (PEG) for increased stability in complex aqueous media (see Additional file 1: Figure S3). These AuNP@PEG were then functionalized with thiol-oligo-fluorophore hairpins: approximately 63 hairpins labeled with the Cy3 donor for BioCode-e13 and 93 hairpins labeled with FAM for BioCode-e14. The UV–Vis spectra for the Au-nanobeacons show a decrease in the SPR frequencies and a broader full-width-at-half-maximum. Dynamic light scattering (DLS) and zeta potential (ζ-potential) of both constructs are similar and higher than the citrate caped AuNP. Together, these data support a successful functionalization—see Additional file 1: Figures S4 and S5 for full characterization. Both BioCodes were washed until no fluorescence was detected on the supernatants to ensure that there are no free donors labeled hairpins in solution, i.e. after the washing steps, all the donor labeled oligonucleotides still in solution are those bound to the AuNPs’ surface.

### FRET based Au-nanobeacon target detection

The BioCode Au-nanobeacon performance was assessed for different experimental conditions: (1) absence of target (reaction blank); (2) non-complementary target (negative reaction); (3) oligonucleotides harboring either the e13a2 or the e14a2 fusion site, depending on the Au-nanobeacon (positive reaction); and (4) absence of target and acceptor (donor blank). The donor blank provides the highest fluorescence intensity of the donor due to minimization of nanoparticle surface energy transfer (NSET [25]) and in the absence of FRET (energy transfer from the donor to the acceptor labeled oligonucleotide); whereas the reaction blank and negative reaction indicate the basal fluorescence of the assay in absence of FRET. This calibration is of paramount relevance considering the cross excitation of the acceptors at the excitation wavelength of the donor, which needs to be “corrected” to allow for clear signal output [16].

**Fig. 1:**
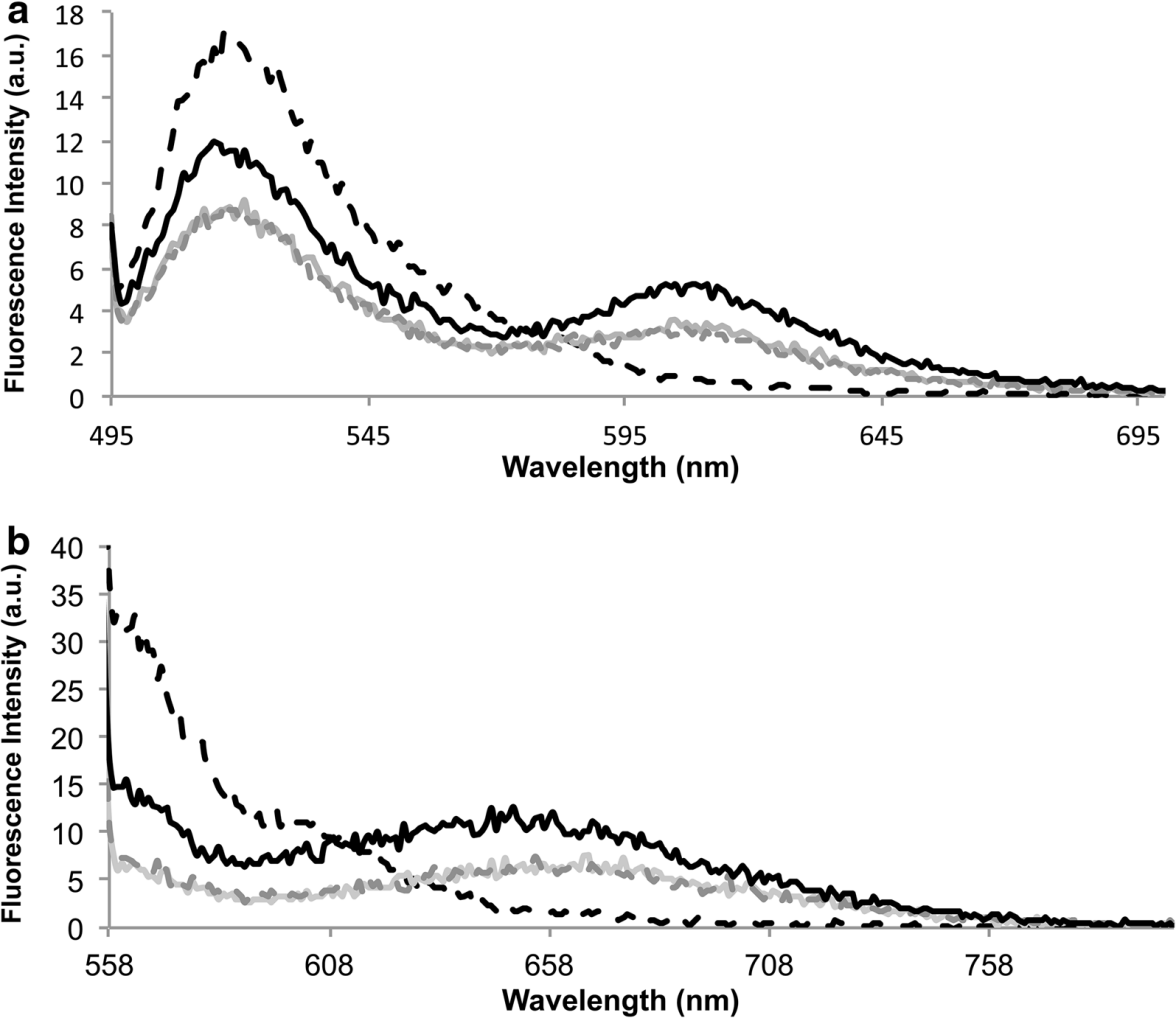
**a** Emission spectra after hybridization in different reaction condition using BioCode-e13. **b** Emission spectra after hybridization in different reaction condition using BioCode-e14. Emission spectra of donor blank reaction (*dashed black line*); positive reaction (*solid black line*); reaction blank (*solid light grey line*); negative reaction (*dashed dark gray line*)

Fluorescence emission spectra for all conditions were collected after the hybridizations events (see experimental section for details). Upon excitation at 495 (donor FAM—BioCode-e13) or at 550 nm (donor Cy3—BioCode-e14), the donor/acceptor pair formed in solution is revealed through the increased fluorescence of the respective acceptor (ROX or Dy-520XL mega Stokes) at 605 and 650 nm, respectively. These spectra were compared to the control reactions (Fig. 1a, b).

Figure 1a shows the emission spectra of BioCode-e13 designed to detect the fusion sequence e13a2, where the FRET signal of the Au-nanobeacon hybridized to the fully complementary target (solid black line) presents the highest fluorescence intensity. Here, the donor is displaced from the surface of the AuNP (lower NSET quenching) and free from the presence of the acceptor (not quenched by FRET). In absence of target (reaction blank—solid light gray) or in presence of a non-complementary target (reaction negative-dark grey line), donor quenching is evident (closed loop conformation). In this situation, the background emission at 605 nm results from the direct excitation of the acceptor at 495 nm, since no opening of the hairpin occurred. When the complementary target and acceptor labeled oligonucleotide are present in solution (positive reaction—dashed black line), both the donor and acceptor increase their emission bands when comparison to the previously described situation. The donor emission in the positive reaction increases due to the increase in distance with respect to the AuNP surface but suffers a decrease due to the energy transfer to the acceptor and, therefore, does not reach values as high as for the donor blank. Since all fluorophore and NP concentrations are kept the same, FRET shows a clear signature on the emission increase at 605 nm, with respect to the direct excitation emission observed in the controls. Therefore, the partial recovery of fluorescence of the donor coupled to the increase of the fluorescence of the acceptor is a dual channel specific hybridization spectral signature. This presents a crosscheck confirmation over the same hybridization event. The same behavior can be observed for BioCode-e14 designed to detect the e14a2 sequence—Fig. 1b.

**Fig. 2:**
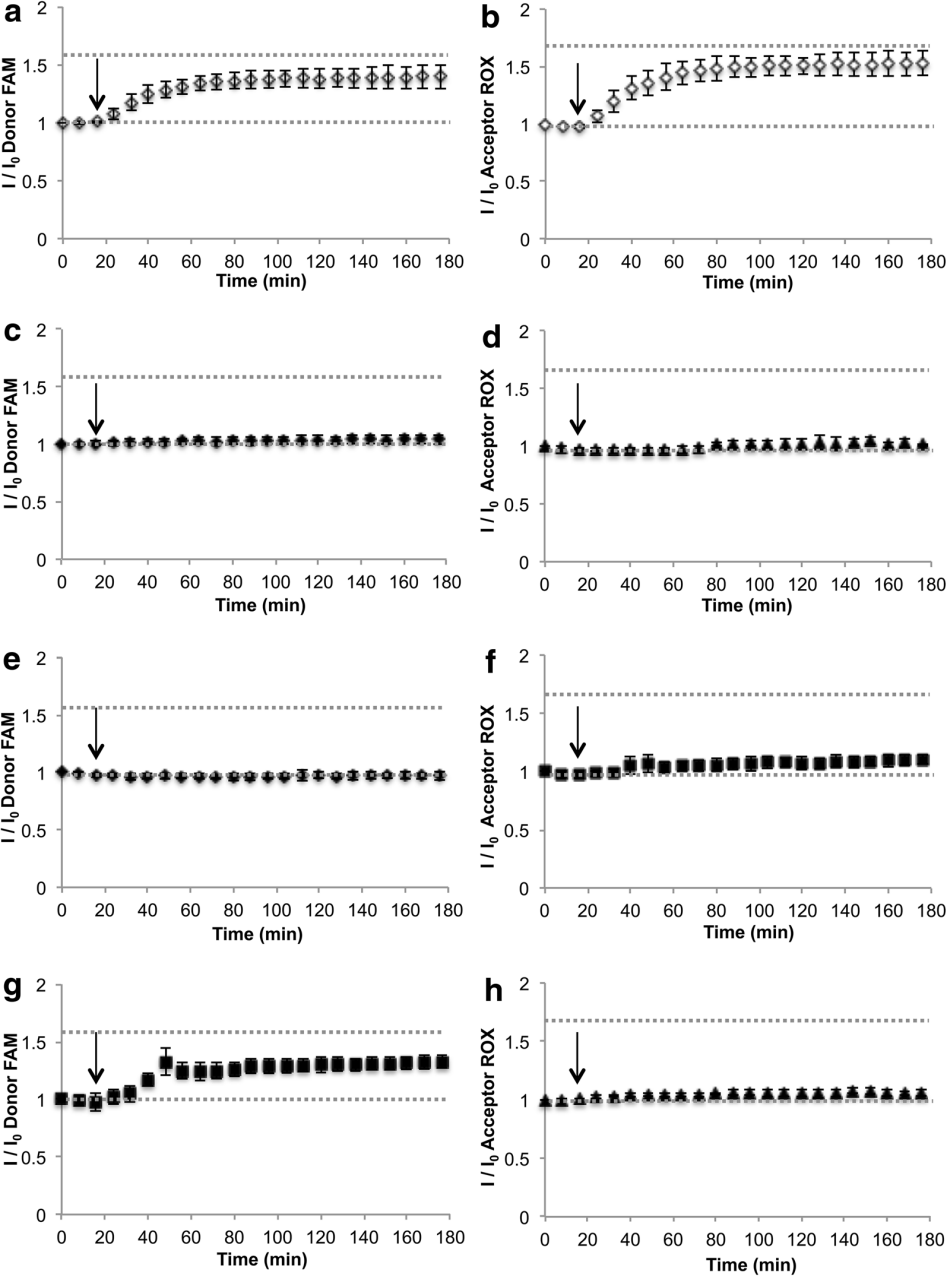
Hybridization assays of BioCode in presence of different target sequences. **a** BioCode-e13 donor emission in the presence of e13a2 complementary target (*white diamonds*); **b** acceptor-ROX emission in the presence of e13a2 complementary target; **c** BioCode-e13 donor emission in the presence of non-complementary target (*black diamonds*); **d** acceptor-ROX emission in the presence of non-complementary target (*black diamonds*); **e** BioCode-e13 donor emission in the presence of exon 13 BCR derived target (*black squares*); **f** acceptor-ROX emission in the presence of exon 13 BCR derived target (*black squares*); **g** BioCode-e13 donor emission in the presence of ABL target (*black diamonds*); **h** acceptor-Dy emission in the presence of ABL target (*black diamonds*). The *black arrow* represents the addition of the target sequence

The dual channel spectral signature—BioCode—was used to follow the hybridization process over time. The channels were defined as follows: (1) donor channel is the integrated emission between 506 and 560 nm (for donor FAM—BioCode-e13) or the integrated emission between 559 and 600 nm (for donor Cy3—BioCode-e14); (2) acceptor channel is the integrated emission from 600 to 700 nm (Acceptor-ROX) or 650–800 nm (Acceptor-Dy); the acceptors limits were set to minimize contribution from donor emission. All data was normalized to the fluorescence signal prior to the addition of target (I_0_). For BioCode-e13, the evolution of the emission channels upon addition of BCR-ABL—Fig. 2a, b, non-complementary target (Fig. 2c, d), BCR (Fig. 2e, f) and ABL (Fig. 2g, h) show that only the fully complementary target leads to the simultaneous increase in both channels. Addition of BCR induces a slight a change in the signal of the donor channel but no significant variation in the acceptor channel, showing that the use of two channels increases detection selectivity. The same behavior is observed for the BioCode-e14 (see Additional file 1: Figure S6).

Considering that CML presents itself in heterozygosity, any given individual will always harbor one healthy copy of ABL and one of BCR. This way, BioCode should be able to differentiate between a full- and a non-complementary target, whilst sustaining the influence of the partial complementary targets. Table 1 summarizes the fluorescence signal percentage for both channels for both BioCode after the addition of a non-complementary, fully complementary and partially complementary target (BCR or ABL) for the most common BCR-ABL fusion sequences (e13a2 and e14a2).

**Table 1:** Percentage variation after target addition for the tested condition

	Reaction blank (%)	ABL (%)	BCR (%)	Negative (%)	Positive (%)
Donor					
BioCode-e13	0.5	5.7	33.6	4.0	39.8
BioCode-e14	8.9	18.2	26.3	1.6	135.6
Acceptors					
BioCode-e13	−8.4	1	10.3	−2.8	53.2
BioCode-e14	−2.6	0.8	1.1	4.5	50.9

The percentage variation of the donor signal for both BioCode (after hybridization) shows a clear difference between the positive reaction (presence of a fully complementary target), negative reaction (presence of a non-related target) and the ABL reaction (partially complementary target). For BioCode-e13, the presence of the BCR portion derived from the e13a2 fusion sequence generates a donor signal that is comparable with the positive reaction (BCR-e13—33.6 % vs. positive—39.8 %, respectively). Despite the signal originating from the partial hybridization of BCR to BioCode-e13, a clearer difference can be observed on the acceptor channel (positive—53.2 % vs BCR-e13—10.3 %). This illustrates the importance of this second channel (acceptors emission) for the confirmation over the same hybridization event. This second channel profits from the wavelength shift mediated by FRET, where the donor’s excitation energy is transferred to an acceptor that does not emit at a wavelength overlapping the AuNPs’ LSPR.

For BioCode-e14, the presence of the BCR sequence induces an increase of 26.3 % on the donor channel (also in the presence of the ABL sequence—18.2 %), whereas the positive reaction induces a 135.6 % increase, allowing clear differentiation of both situations. Again, the signal variation on acceptor channel is residual for the ABL and BCR situations (0.8 and 1.1 %, respectively), while the positive reaction shows a clearly higher signal variation (50.9 %). See also Additional file 1: Figure S5 for time-lapse hybridization of BioCode-e14.

The BioCode Au-nanobeacon concept may be easily expanded to further targets, allowing for the evaluation of complex traits resulting from combination of the expression of several loci.

## Conclusions

We present a novel concept—BioCode Au-nanobeacons—suitable for the selective detection of similar targets in solution. This FRET based Au-nanobeacon system was applied to the selective detection of specific molecular targets that are of clinical interest for real life situations. As proof-of-concept, we designed the BioCode Au-nanobeacons to detect either one of the most common fusion sequences causing chronic myeloid leukemia, e13a2 and e14a2. The BioCode is a single-step detection strategy based on a two successive hybridization events: hybridization of the target molecule opens the nanobeacon and allows for partial recovery of the donor’s emission and exposes the palindrome sequence that is the target for the acceptor labeled oligonucleotide. The FRET signal is generated solely under selective recognition of the complimentary target, overlooking any signal originated by the presence of partially complementary targets. This is of utmost relevance since unequivocal detection for similar sequences, such as CML, must exclude the unwanted hybridization events resulting from healthy ABL or BCR expressed mRNAs that are always present in any given sample. The cross-reactivity of the healthy counterparts is the most common reason for false positive results in current molecular characterization methods for CML.

## Methods

All reagents were of analytical grade and purchased from Sigma Aldrich. All oligonucleotides were purchased from STABVIDA (Portugal) and used without further purification unless stated otherwise.

### Fluorophore selection

Donor and acceptor fluorophores were chosen based on their spectroscopic characteristics: (1) the donor emission band should overlay within the LSPR of the AuNP; (2) the acceptor’s absorption band should maximize the overlap with the donor’s emission; and (3) the emission maxima of the acceptors should be distinct to allow identification of the different emission bands while minimizing its overlap with the LSPR. From the commercially available fluorophores, we selected 6-carboxifluorescein (FAM) as donor for BioCode-e13 and sulfoindocyanine Cy3 (Cy3) as donor for BioCode-e14; 5-carboxy-X-rhodamine (Acceptor-ROX) as the acceptor for FAM and Dy-520XL mega Stokes (Acceptor-Dy) as the acceptor for Cy3. See Additional file 1: Figures S7 and S8 for full absorption and emission spectra of these fluorophores.

### Hairpin design and target sequences

Au-nanobeacons were designed and the hairpin optimized to specifically detect the BCR-ABL fusion region of e13a2 and e14a2 transcript sequence (Accession no. AJ131467.1 and AJ131466.1, respectively). The synthetic oligomer targets were designed considering several scenarios: (1) complementary target (e13a2 or e14a2); (2) unrelated non-complementary target, originating from an unrelated sequence (NC); (3) one sequence harboring a partial region derived from exon 2 of the ABL gene (ABL); (4) one sequence harboring a partial region derived from exon 13 of the BCR gene (BCR-e13); (5) one sequence harboring a partial region derived from exon 14 of the BCR gene (BCR-e14); (6) acceptor sequence complementary to the palindrome sequence of the hairpin constituting each nanobeacon. See Table 2 for sequences.

**Table 2:** Oligonucleotide sequences used for AuNP functionalization, target specificity and acceptor labeled oligonucleotides

	Oligonucleotide sequence (5′–3′)	5′ modification	3′ modification
Hairpin-e13a2	ccacgccaaacgctgaagggcttcttccttatttttggcgtgg	C_6_-Thiol	6-carboxyfluorcein
Hairpin-e14a2	cacctcgaaatctgaagggcttttgaactctgttttcgaggtg	C_6_-Thiol	Cy3
e13a2 (AJ131467.1)	ataaggaagaagcccttcagcg	–	–
e14a2 (AJ131466.1)	cagagttcaaaagcccttcag	–	–
Acceptor-ROX	ccacgccaaa	–	ROX
Acceptor-Dy	cacctcgaaa		Dy-520XL mega stokes
BCR-e13 (NM_004327.3)	tccgctgaccatcaataaggaagaa	–	–
BCR-e14 (NM_004327.3)	cactggatttaagcagagttcaaaa	–	–
ABL (NM_005157.5)	gcccttcagcggccagtagcatctg	–	–
Non-complementary	attaccagacatgcgtggtcccaac	–	–

### Evaluation of target selectivity of the designed hairpins

Hybridization of the hairpins to their respective target sequence and cross hybridization was evaluated using 0.5 µM of each hairpin (either e13a2 or e14a2, both marked with FAM as donor) or 0.25 µM using both hairpins. Reactions were performed in 0.5 × TBE pH 8, 154 mM NaCl using 1 µM of each target and 0.5 µM each acceptor (acceptor-ROX for the e13a2 hairpin; acceptor-Dy for the e14a2 hairpin; or both acceptors for both hairpins). The positive sample contains the complementary target to the respective hairpin; the negative sample contains a non-complementary target for both hairpins; the ABL reaction contains a target with the ABL sequence (common portion of the BCR-ABL fusion product); the BCR contains a target with the BCR sequence of each fusion sequence (containing either the e14 or e13 portion of the BCR-ABL fusion product); the BCR + ABL reaction contains both the non-fused BCR and ABL templates; the cross-template reaction contains either the e13a2 template for the e14a2-hairpin or the e14a2 template for the e13a2-hairpin.

### Gold nanoparticle synthesis

Gold nanoparticles with an average size of 14 nm were synthesized using the citrate reduction method by Turkevich [26] and adapted by Lee and Miesel [27]. Briefly, 250 ml solution of HAuCl_4_ at 1 mM was heated until boiling with continuous stirring. Upon reflux, 25 ml of sodium citrate at 38.8 mM was quickly added to the HAuCl_4_ solution, left refluxing for 20 min under continuous stirring protected from light, and cooled down to room temperature before use. Transmission electron microscopy (TEM), dynamic light scattering (DLS) and UV–Vis spectroscopy were used to characterize the synthetized AuNPs.

### BioCode Au-nanobeacon assembly

The BioCode Au-nanobeacons were synthesized as described in [10]. Briefly, AuNPs were functionalized for 45 % surface coverage with polyethylene glycol (PEG) using a commercial hetero-functional PEG modified with a thiol group O-(2-mercaptoethyl)-O’-methyl-hexa(ethylene glycol), C_15_H_32_O_7_S, 356.48 Da. The PEGylated AuNPs (AuNP@PEG) were washed by centrifugation (14,000×*g*, 45 min, 4 °C). For Au-nanobeacon conjugation, the stem-looped oligonucleotide modified with 3′-FAM and 5′-Thiol-C6 (STABVIDA) was suspended in 1 mL of 0.1 M dithiothreitol (DTT), extracted three times with ethyl acetate and further purified through a desalting NAP-5 column (Pharmacia Biotech) using 10 mM phosphate buffer (pH 8) as eluent. The oligonucleotide was added to the PEGylated AuNP in a 100:1 ratio and processed as previously described [10]. The Au-nanobeacons were centrifuged at 14,000×*g* for 45 min, 4 °C, the pellet washed 3 times, and re-suspended in 10 mM phosphate buffer (pH 8), for a final concentration of 20 nM. The resulting Au-nanobeacons were stored in the dark at 4 °C until further use. The BioCode Au-nanobeacons were characterized by DLS, UV–Vis spectroscopy and zeta potential. Supernatants were collected for determination of number of oligonucleotides per nanoparticle.

### Fluorescence analysis

Fluorescence emission spectra were collected on a Varian Cary Eclipse Fluorescence Spectrophotometer with 5 nm bandwidth excitation and emission slits in a 3 mm optical path quartz cuvette (HELLMA, Germany). The excitation wavelength used was 495 nm (maximum absorption of FAM—donor) and 550 nm (maximum absorption of Cy3). Fluorescence measurements were performed at 20 °C. The occurrence of energy transfer (FRET) was determined through the increase of the intensity of fluorescence band of the acceptors compared to the intensity of the fluorescence band of the acceptors in the control reaction.

### Detection of target sequence

All detection/hybridization assays were performed with 1 nM of Au-nanobeacon in 0.5 × TBE pH 8, 154 mM NaCl. For the Positive sample (POS), 500 nM of e13a2 or e14a2 target sequence and 50 nM of the respective acceptor; for the Negative (NEG), 500 nM of NC target and 50 nM of the respective acceptor; for Donor blank, 500 nM of complementary target and with 50 nM of the respective acceptor; for reaction blank water was added to the reaction in the presence of 50 nM of the respective acceptor.

To control and fully characterize the detection events, hybridizations were performed by (1) pre-hybridize the respective targets before collecting the emission spectra (95 °C for 30 s and 20 °C for 30 min); (2) collecting several emission spectra before and after of addition of the respective targets and follow hybridization over time.

## Additional file

10.1186/s12951-016-0192-y In silico verification of hybridization of the designed sequences using the software NUPACK. **Figure S2**. Schematic representation of specific target recognition by the designed hairpins. **Figure S3**. Experimental validation of hybridization of the designed sequences. **Figure S4**. Calibration of PEG functionalization of AuNP surface. **Figure S5**. Characterization of the synthetized BioCodes and gold nanoparticles. **Figure S6**. Hybridization assays of BioCode e14. **Figure S7**. Normalized absorption and emission spectra of the fluorophores used for BioCode-e13 and absorption spectra of AuNP. **Figure S8**. Normalized absorption and emission spectra of the fluorophores used for BioCode-e14 and absorption spectra of AuNP.

## Abbreviations

AuNP: gold-nanoparticle
LSPR: localized surface plasmon resonance
FRET: Forster resonant energy transfer
CML: chronic myeloid leukemia
