# Supplementary material for: BioCode gold-nanobeacon for the detection of fusion transcripts causing chronic myeloid leukemia
Source: J Nanobiotechnology. 2016 May 17;14:38. doi: 10.1186/s12951-016-0192-y (PMC4869199; doi:10.1186/s12951-016-0192-y)
Supplement: Supplementary file 1 — 10.1186/s12951-016-0192-y In silico verification of hybridization of the designed sequences using the software NUPACK. Figure S2. Schematic representation of specific target recognition by the designed hairpins. Figure S3. Experimental validation of hybridization of the designed sequences. Figure S4. Calibration of PEG functionalization of AuNP surface. Figure S5. Characterization of the synthetized BioCodes and gold nanoparticles. Figure S6. Hybridization assays of BioCode e14. Figure S7. Normalized absorption and emission spectra of the fluorophores used for BioCode-e13 and absorption spectra of AuNP. Figure S8. Normalized absorption and emission spectra of the fluorophores used for BioCode-e14 and absorption spectra of AuNP. [file 12951_2016_192_MOESM1_ESM.pdf]

**BioCode Gold-Nanobeacon for the detection of fusion transcripts causing chronic myeloid leukemia**

M. Cordeiro [1,2], L. Giestas [1,2], J. C. Lima[1] and P. M. V. Baptista[2, *]

[1]LAQV, REQUIMTE, Departamento de Química, Faculdade de Ciências e Tecnologia, Universidade Nova de Lisboa, Campus de Caparica, 2829-516 Caparica, Portugal;

[2]CIGMH, UCIBIO, Departamento de Ciências da Vida, Faculdade de Ciências e Tecnologia, Universidade Nova de Lisboa, Campus de Caparica, 2829-516 Caparica, Portugal;

* Corresponding Author. Email: pmvb@fct.unl.pt; Phone/Fax: +351 21 294 8530.

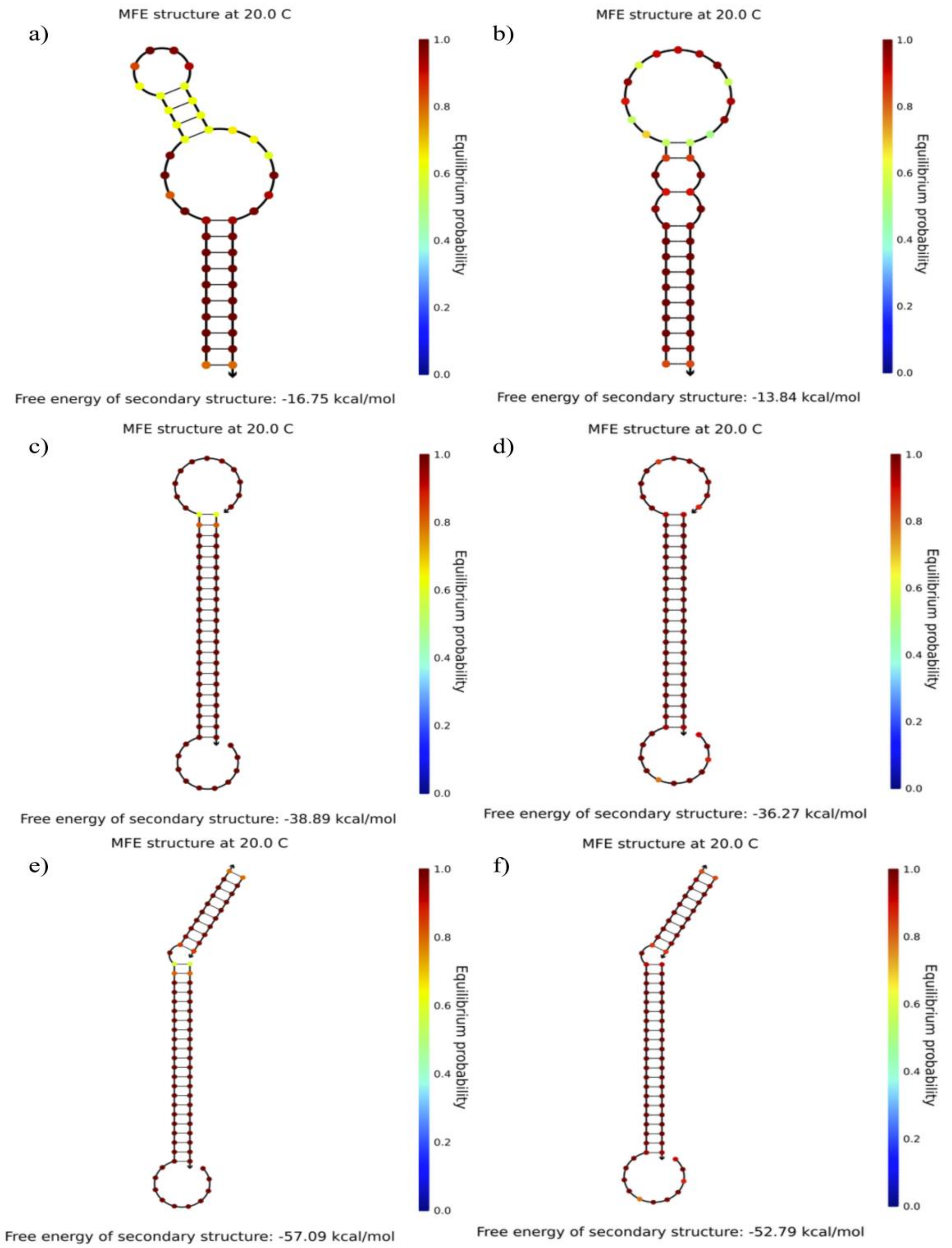


**Figure S1.** *in silico* verification of hybridization of the designed sequences using the software NUPACK; a) e13a2-Hairpin in absence of target; b) e14a2-Hairpin in absence of target; c) e13a2-Hairpin in the presence of complementary target; d) e14a2-Hairpin in presence of complementary target; e) e13a2-Hairpin in presence of complementary target and of complementary acceptor labeled oligonucleotide; f) e14a2-Hairpin in presence of complementary target and of complementary acceptor labeled oligonucleotide.

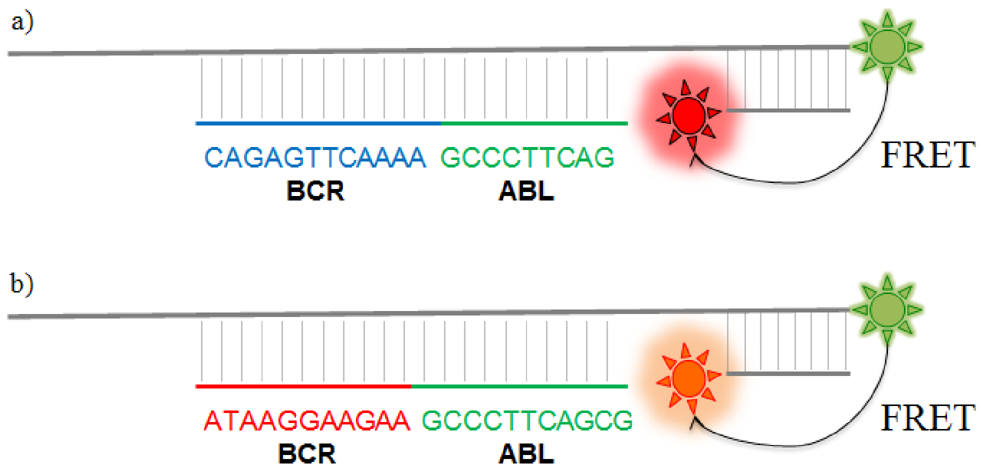


**Figure S2.** Schematic representation of specific target recognition by the designed hairpins; a) e14a2-hairpin hybridized to the e14a2 target; b) e13a2-hairpin hybridized to the e13a2 target;

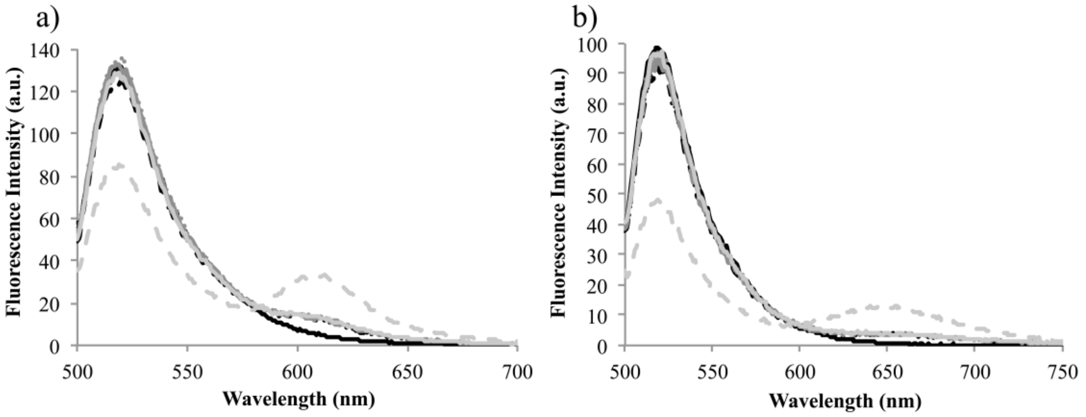


**Figure S3.** Experimental validation of hybridization of the designed sequences; a) e13a2-hairpin characterization; b) e14a2-hairpin characterization. Emission spectra of: donor blank reaction (solid black line); reaction blank (dashed black line); non-complementary reaction (dotted black line); ABL reaction (solid dark grey); BCR reaction (dashed dark grey line); BCR+ABL reaction (dotted dark grey line); cross template reaction (solid light grey line); positive reaction (dashed light grey line).

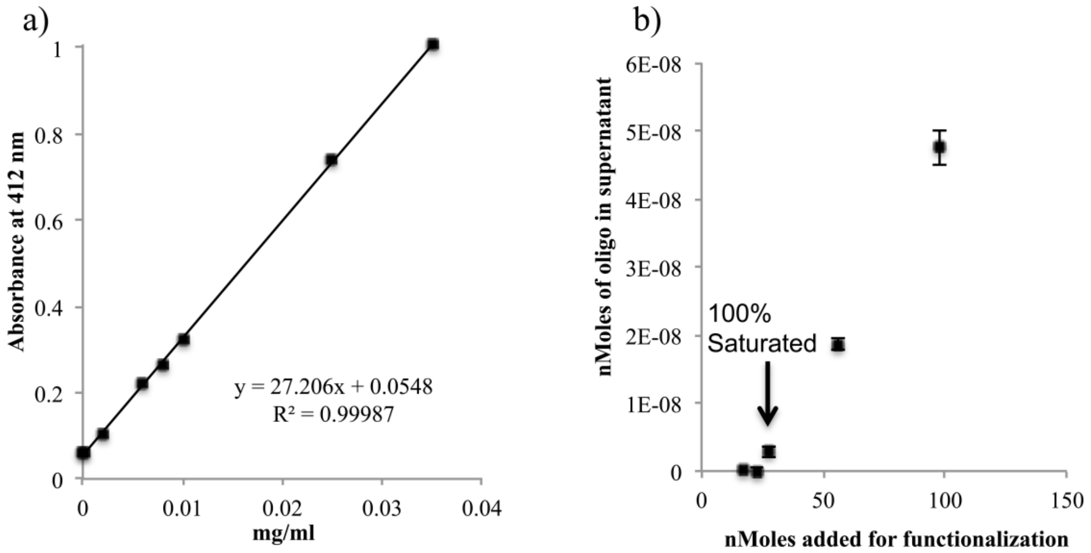


**Figure S4.** Calibration of PEG functionalization of AuNP surface.
a) Calibration curve used for the quantification of thiolated PEG in the supernatant; b) Quantification of PEG in supernatant as a function of the added PEG, corrected for the residual absorbance of DTNB. The error bars represent 3 independent assays;

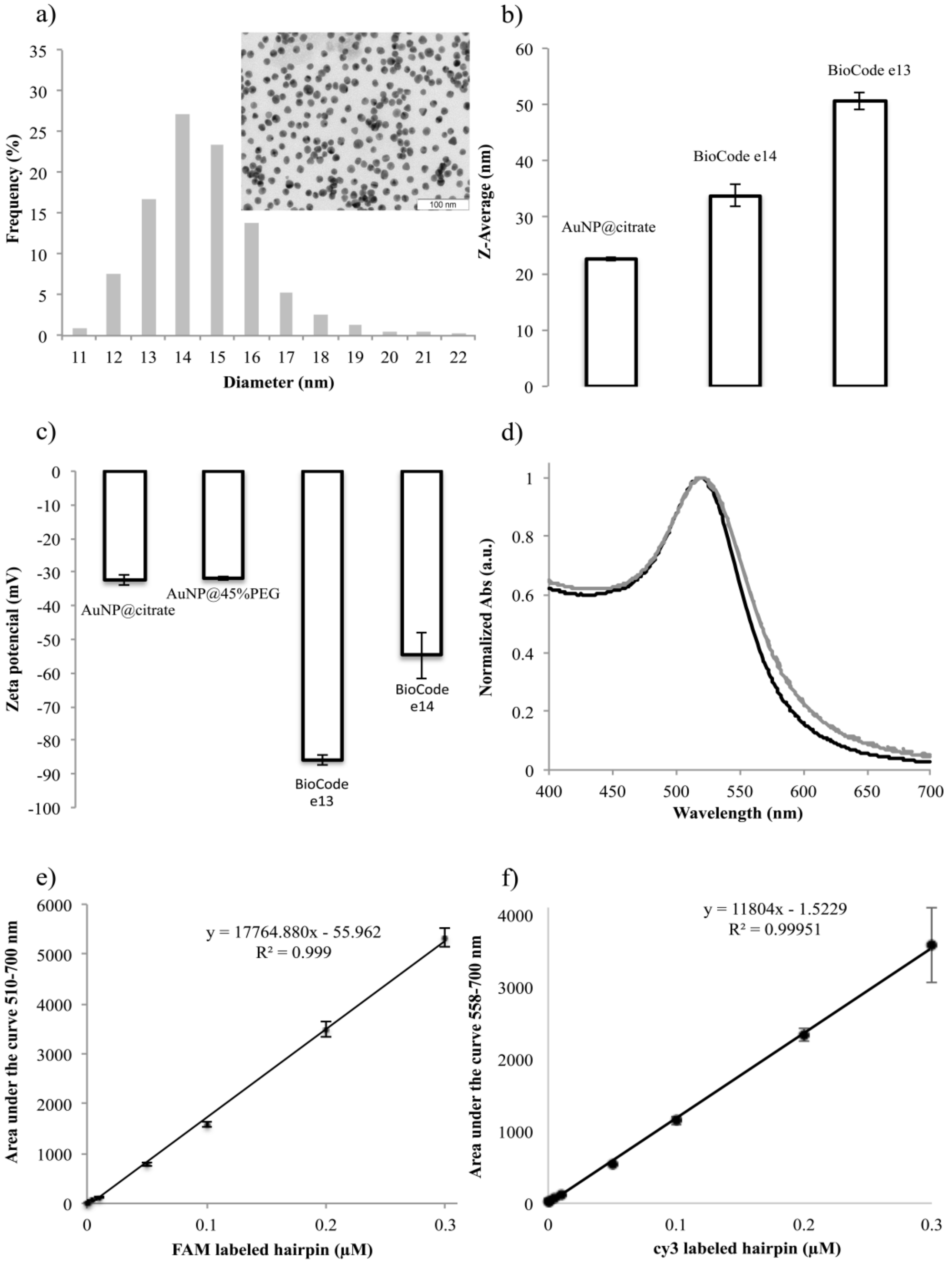


**Figure S5.** Characterization of the synthetized BioCodes and gold nanoparticles;
a) Size distribution of the synthetized AuNP, Inset: TEM image of the AuNP (scale bar: 100nm); b) Hydrodynamic diameter of the AuNP, BioCode-e13 and BioCode-e14; c) zeta potential of citrate capped AuNP, PEG and Au-nanobeacons d) UV-VIS spectra of the AuNP (solid black line), BioCode-e13 (solid grey line) and BioCode-e14 (dashed grey line); e) calibration curve for the quantification of hairpins per AuNP for the BioCode-e13; f) calibration curve for the quantification of hairpins per AuNP for the BioCode-e14; error bars correspond to 3 independent assays

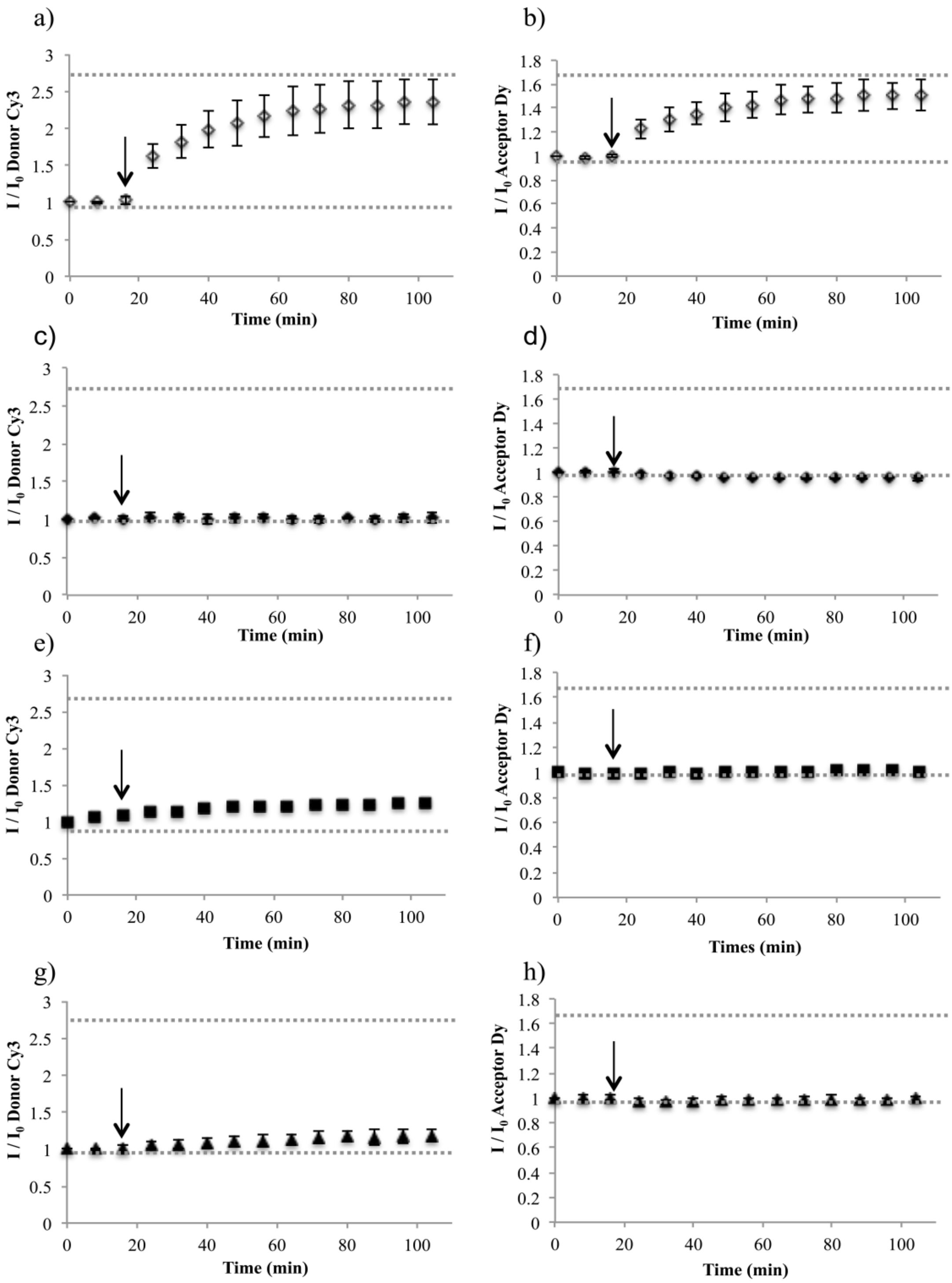


**Figure S6.** Hybridization assays of BioCode e14.
a) BioCode-e14 donor emission in presence of e14a2 complementary target (white diamonds); b) Acceptor-Dy emission in presence of e14a2 complementary target; c) BioCode-e14 donor emission in presence of non-complementary target (black diamonds); d) Acceptor-Dy emission in presence of non-complementary target (black diamonds); e) BioCode-e14 donor emission in presence of exon 14 BCR derived target (black squares); f) Acceptor-Dy emission in presence of exon 14 BCR derived target (black squares) g) BioCode-e14 donor emission in presence of ABL target (Black diamonds); h) Acceptor-Dy emission in presence of ABL target (Black diamonds). The error bars represent at least 3 independent assays. The black arrow represents the addition of the target sequence.

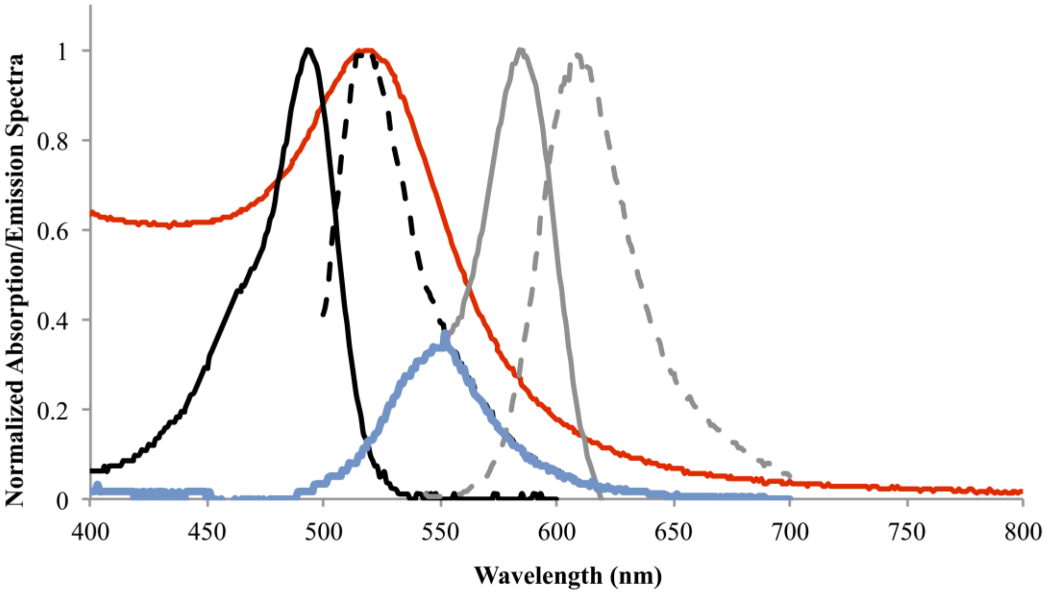


**Figure S7**. Normalized absorption and emission spectra of the fluorophores used for BioCode-e13 and absorption spectra of AuNP.

Solid black line –FAM Absorption, dashed black line- FAM emission, solid grey line –ROX Absorption, dashed grey line –ROX Emission; Solid red line- AuNP Absorption; Solid blue line – overlap between emission spectra of FAM and absorption spectra of ROX;

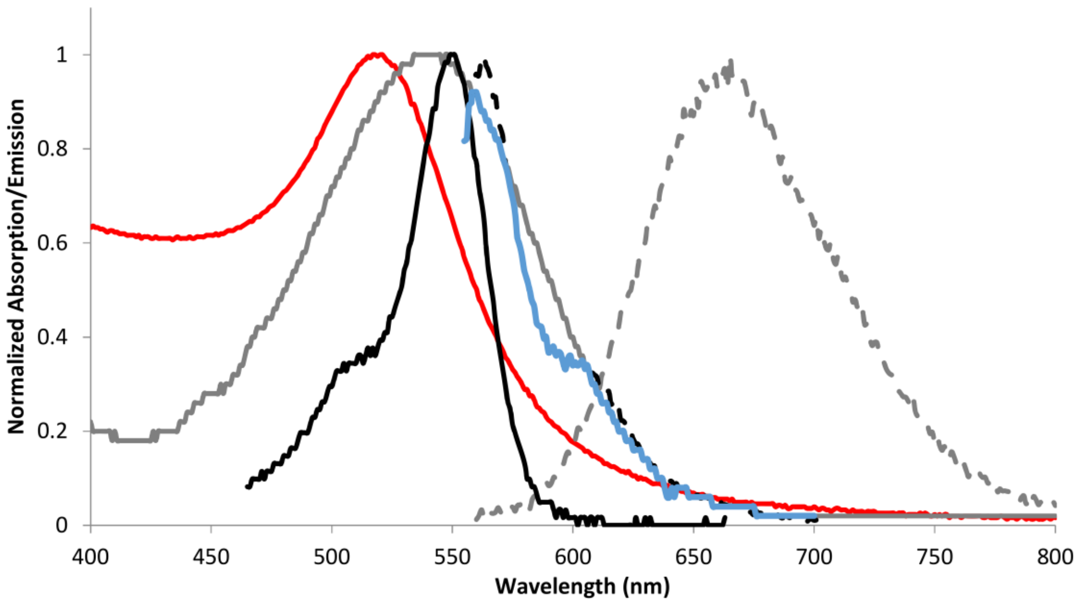


**Figure S8**. Normalized absorption and emission spectra of the fluorophores used for BioCode-e14 and absorption spectra of AuNP.

Solid black line – Cy3 Absorption, dashed black line – Cy3 emission, solid grey line – Dy-520XL megastockes Absorption, dashed grey line – Dy-520XL megastockes Emission; Solid red line- AuNP Absorption; Solid blue line – Overlap between emission spectra of Cy3 and absorption spectra of Dy-520XL megastockes;
